# The replisome guides nucleosome assembly during DNA replication

**DOI:** 10.1186/s13578-020-00398-z

**Published:** 2020-03-12

**Authors:** Wenshuo Zhang, Jianxun Feng, Qing Li

**Affiliations:** State Key Laboratory of Protein and Plant Gene Research, School of Life Sciences and Peking-Tsinghua Center for Life Sciences, Peking University, Beijing, 100871 China

**Keywords:** Replisome component, Nucleosome assembly, Chromatin replication, Histone chaperone

## Abstract

Nucleosome assembly during DNA replication is tightly coupled to ongoing DNA synthesis. This process, termed DNA replication-coupled (RC) nucleosome assembly, is essential for chromatin replication and has a great impact on both genome stability maintenance and epigenetic inheritance. This review discusses a set of recent findings regarding the role of replisome components contributing to RC nucleosome assembly. Starting with a brief introduction to the factors involved in nucleosome assembly and some aspects of the architecture of the eukaryotic replisome, we discuss studies from yeast to mammalian cells and the interactions of replisome components with histones and histone chaperones. We describe the proposed functions of replisome components during RC nucleosome assembly and discuss their impacts on histone segregation and implications for epigenetic inheritance.

## Background

### A brief introduction to DNA replication-coupled (RC) nucleosome assembly

Eukaryotic DNA replication occurs in the context of the chromatin environment [1]. Chromatin, the carrier of genetic and epigenetic information and guardian of genome stability, must be duplicated in daughter cells to ensure continuity between generations. As the fundamental step of this process, chromatin replication is highly regulated across all species [2–4]. The nucleosome, as the basic unit of chromatin, is composed of a histone octamer containing a H3-H4 tetramer and two H2A-H2B dimers wrapped by approximately 147 base pairs of DNA [5]. During DNA replication, nucleosomes ahead of the replication fork must be disassembled to facilitate the movement of the DNA replication machinery, and behind the fork, new nucleosomes must be reformed on daughter strands with both recycled parental histones and newly synthesized histones to restore the chromatin state. This process, called DNA replication-coupled (RC) nucleosome assembly, is an essential step for chromatin replication [2, 4, 6].

Nucleosome assembly during DNA replication occurs in a stepwise fashion. Early studies using a chemical cross-linking technique combined with radioisotope labeling methods demonstrated that parental histone H3–H4, which is one of the major carriers of epigenetic information, needs to be recycled during replication [7–9]. Recently, using SILAC (stable isotope labeling by amino acids in cell culture)-based quantitative mass spectrometry, it was established that histone H3–H4 tetramers are mainly recycled in a non-split fashion during DNA replication [10]. Therefore, H3–H4 is deposited first in tetrameric form, followed by the rapid deposition of two H2A–H2B dimers, thus forming an intact nucleosome [3, 11–13]. Whether H2A–H2B is recycled during replication is less clear, partially due to their highly dynamic features [14–16]. The histone H3–H4 tetramers can form stable intermediates with DNA called tetrasomes under physical conditions in vitro*,* a process that is facilitated by other factors or happens spontaneously [17]. Thus, the formation of tetrasomes is regarded as the first step of nucleosome assembly. In this review, we mainly focus on the discussion of this step during DNA replication.

Parental histones provide only one-half of the total histone supply, indicating that the other half comprises newly synthesized histones deposited onto daughter strands. It is reported that many factors that regulate the deposition of new histones have been identified [2, 18]. Moreover, accumulated studies demonstrate that histone modifications regulate the histone chaperone-mediated deposition of newly synthesized histones. In general, newly synthesized histone H3–H4 tends to be acetylated [19, 20]. In mammals, newly synthesized histone H3.1–H4 is acetylated by HAT1-RbAp46 acetyltransferase on lysine residues 5 and 12 of H4 (H4K5, 12ac) [19, 21–23]. In yeast, in addition to acetylation on histone H3-H4 tails, the newly synthesized histone H3–H4 is acetylated by Rtt109 acetyltransferase on lysine 56 of H3 (H3K56ac) [20, 24–26]. Both H4K5, 12ac and H3K56ac increase the interactions of histone chaperones with newly synthesized H3–H4 and promote histone chaperone-mediated RC nucleosome assembly [19, 27, 28].

### Histone chaperones are the major players in RC nucleosome assembly

Histones are a group of highly basic proteins. While the basic charges of histones can be neutralized by the phosphodiester backbone of the DNA in the nucleosome particle, histones that are free from chromatin require accompaniment of a group of acidic proteins to prevent aberrant aggregate formation. The proteins dedicated to this group are called histone chaperones, and they play key roles during nucleosome assembly [29, 30]. Over the last 3 decades, significant progress has been made in identifying and characterizing the functions of histone H3-H4 chaperones involved in newly synthesized histone H3-H4 deposition, such as CAF-1 (chromatin assembly factor-1) [31], Asf1 (anti-silencing factor 1) [32], Rtt106 (regulator of Ty1 transposon 106) [27, 33], and FACT (facilitates chromatin transaction) [34].

The histone chaperone Asf1 is shown to facilitate H3-H4 import into the nucleus via importin proteins in the nuclear pore complex [35, 36]. In budding yeast, after entering the nucleus, Rtt109/Vps75 acetylates lysine 56 on H3 only when H3 is bound to Asf1 [24–26, 37, 38]. Asf1 has been shown to bind H3-H4 dimers and to block H3-H4 tetramer formation [39–41]. In addition, Asf1 has not been shown to be active in the assembly of histones on DNA during the formation of nucleosomes in vitro, indicating that Asf1 may not participate in the assembly event directly [41, 42]. Accumulated evidence suggests that Asf1 delivers histone H3-H4 dimers to other histone chaperones, such as CAF-1 and Rtt106 [43–45]. Asf1 can bind the Cac2 subunit of the histone chaperone CAF-1, and conformational changes allow the delivery of H3-H4 dimers from Asf1 to CAF-1, providing direct evidence for coordination between histone chaperones [44–47]. While H3K56ac and H4K5,12ac increase the interaction of CAF-1 and Rtt106 with newly synthesized H3-H4, ubiquitination of H3 at lysine residues 121, 122, and 125 (H3K121, 122, 125ub) decreases the interaction of histone H3-H4 with Asf1, suggesting that histone modifications are important in regulating the histone transfer process [27, 28, 43].

CAF-1 was the first identified DNA replication factor coupled to chromatin assembly [31]. CAF-1 is a heterotrimeric complex composed of three subunits, Cac1, Cac2 and Cac3 (p150, p60 and p48 in human cells). Both the Cac1 and Cac2 subunits are required for binding to H3-H4, while the Cac3 subunit is not required [27, 48]. Cac1 binds histone H3-H4 in one acidic region called the ED domain [49]. The middle part of Cac2 can also bind H3-H4 [50]. Moreover, CAF-1 can bind DNA directly via two domains in the Cac1 subunit called WHD and KER. The results from a structure analysis revealed that the binding of CAF-1 to one H3-H4 dimer abolishes the inhibition of the ED domain to the WHD domain. Therefore, the CAF-1-H3-H4 complex can bind DNA, and the cooperative DNA binding by CAF-1 promotes the rapid dimerization of the CAF-1-H3-H4 complex, thus forming H3-H4 tetramers [50, 51]. Although S phase progression is delayed, deletion of *CAC1* is not lethal [49], suggesting that other histone chaperones are also involved in RC nucleosome assembly.

FACT was initially identified as a factor that facilitates transcription via the disassembly of H2A-H2B [52, 53]. Recently, it was shown that FACT binds both H2A-H2B and H3-H4 and has both nucleosome assembly activity and disassembly activity [54–56]. Moreover, FACT is involved not only in the deposition of newly synthesized H3-H4 but also in the recycling of parental histones during replication [34, 57–59]. In budding yeast, FACT interacts with both CAF-1 and Rtt106, suggesting a collaboration among histone chaperones [34]. Rtt106, a H3-H4 histone chaperone in budding yeast, recognizes H3K56ac and binds new H3-H4 tetramers, contributing to the deposition of new histones [27, 60, 61]. Thus, multiple histone chaperones contribute to the RC nucleosome assembly pathway. For a more detailed description of histone chaperones, we refer the reader to some recent excellent reviews [29, 36, 62].

### Overview of eukaryotic replisome assembly

DNA replication starts at specific DNA elements termed replication origins. In the budding yeast *Saccharomyces cerevisiae*, replication origins contain specific elements called autonomously replicating sequences (ARSs) [63]. To date, metazoan replication origins are not well defined at the DNA sequence level, indicating that chromatin organization might be critical for replication initiation [64, 65]. To ensure the efficiency and high fidelity of DNA replication in eukaryotic cells, the assembly of the replisome at replication forks is critical during DNA replication [66, 67]. Replisome assembly not only links the replicative helicase to DNA polymerases but also incorporates many other factors to ensure highly efficient coordination during the DNA replication process [68, 69].

Eukaryotic replisome assembly is highly conserved from yeast to human cells [70, 71]. In budding yeast, when cells exit the last phase of the cell cycle, ORC (origin recognition complex), containing six subunits (Orc1-6), recognizes ARS elements on chromatin [11, 72, 73]. Upon cells entering the G1 phase, licensing factors Cdc6 (cell division control protein 6) and Cdt1 (Cdc10-dependent transcript 1) load MCM2-7 (minichromosome maintenance complex 2–7) to ORC binding sites to form pre-replication complex (pre-RC) in a process called origin licensing [74, 75]. The MCM2-7 complex is loaded as a head-to-head double hexamer, and after it has been loaded onto DNA, the origin becomes licensed and is ready to be activated [76]. When the cell enters the S phase, two S phase-dependent kinases, DDK (Dbf4-dependent kinase) and CDK (cyclin-dependent kinase), phosphorylate the MCM2-7 complex and other key factors. Then, Cdc45 (cell division control protein 45) and the GINS complex (Sld5, Psf1, Psf2, and Psf3) are recruited to assemble the CMG complex (Cdc45-MCM2-7-GINS), which is the active form of the replicative helicase [77, 78]. It is believed that Mcm10 is also required for DNA replication initiation and for the loading of the DNA polymerase α-primase complex [79–81]. Once activated, helicase unwinds the DNA duplex, resulting in single-strand DNA (ssDNA), which is rapidly bound by RPA (replication protein A) [82]. The homotrimer of Ctf4 (chromosome transmission fidelity 4) serves as a bridge from Pol α to the CMG complex [83]. Primase generates a short RNA primer of approximately 10 nucleotides to provide 3′-OH, and then, the DNA polymerase of Pol α synthesizes 20 to 30 deoxynucleotides downstream of the RNA primer. The ring-shaped sliding clamp in eukaryotes, called PCNA (proliferating cell nucleus antigen), is loaded such that it surrounds the primer-template junctions by the ATP-dependent clamp loader RFC complex (replication factor C) [84]. After PCNA is loaded, the two high processivity DNA polymerases, Pol δ and Pol ε, replace Pol α as the main DNA-synthesizing polymerases on the lagging strand and leading strand, respectively. These two polymerases both have 3′-to-5′ exonuclease (proofreading) activity to guarantee the maximum level of fidelity of DNA synthesis. PCNA dramatically enhances the processivity of the DNA polymerases, especially Pol δ [85]. Due to the 5′-to-3′ direction of DNA synthesis, the leading strand can be replicated continuously, while the lagging strand is synthesized discontinuously in Okazaki fragments. When Pol δ encounters the 5′-end of the previous Okazaki fragment, it moves forward to displace the fragment with a few nucleotides, thus generating a short flap. Then, several endonucleases (Fen1, Dna2 and Exo1 in budding yeast) remove the flap, and the resulted nick is filled by DNA ligase I (Cdc9 in budding yeast). This process is called Okazaki fragment maturation [86]. Recent structural advances and in vitro reconstitution systems have revealed many details of these replisome components, showing them cooperating at replication forks to promote efficient DNA replication [87–91]. Altogether, the replisome assembly is a fundamental process during DNA replication that involves a number of replisome components functioning in a stepwise and highly cooperative fashion.

## Main text

From the disassembly of parental nucleosomes, unwinding of dsDNA, and DNA synthesis to the assembly of daughter nucleosomes, chromatin replication is one of the most complicated molecular events in cells, and it is mediated by an extensive set of protein machinery. To ensure the tight coupling of nucleosome assembly with ongoing DNA replication, this process involves the concerted regulation of diverse histone chaperones in collaboration with replisome components. Accumulated evidence suggests that the replisome components interact with histone chaperones, some of which may even directly interact with histones (Table 1). Those physical interactions are highly beneficial to the coordination of events occurring at the replication fork.

**Table 1:** Summary of the interactions among replisome components, histones and histone chaperones

Replisome proteins	Histone/histone chaperones	Methods used	Species
PCNA	CAF-1	IP: PCNA-CAF-1 physical interaction In vitro pull down: Cac1 (p150) subunit binds PCNA directly via the PIP domain	*Homo sapiens, Saccharomyces cerevisiae*
RFC	Asf1	In vitro pull down: Asf1 binds the Rfc1 subunit directly via the Asf1 N-terminal	*Saccharomyces cerevisiae*
	Rtt106	IP: Rtt106-Elg1-RFC physical interaction	*Saccharomyces cerevisiae*
RPA	FACT	In vitro pull down: Pob3-M domain binds RPA directly	*Saccharomyces cerevisiae*
	H3–H4/FACT, CAF-1, Rtt106	In vitro pull down: RPA binds H3-H4 directly. RPA binds FACT, CAF-1 and Rtt106 directly	*Saccharomyces cerevisiae*
Pol1 (Pol α)	H2A–H2B	IP: Yeast Pol1-(H2A–H2B) physical interaction In vitro pull down: N-terminal of human and yeast Pol1 has an H2A-H2B-binding domain	*Saccharomyces cerevisiae, Homo sapiens*
	FACT	IP: Pol α-FACT physical interaction In vitro pull down: Pol1 binds FACT directly	*Saccharomyces cerevisiae*
Dpb3-Dpb4 (Pol ε)	H2A–H2B	IP: Yeast Dpb3-Dpb4 has physical interaction with H2A-H2B and H3-H4	*Saccharomyces cerevisiae, Schizosaccharomyces pombe*
	H3–H4	Structural analysis and in vitro pull down: Dpb3-Dpb4 binds H3–H4 directly	*Homo sapiens, Saccharomyces cerevisiae, Schizosaccharomyces pombe*
Mcm2 (MCM2-7)	H3–H4	IP: Mcm2-(H3–H4) physical interaction In vitro pull down: N-terminal terminus of Mcm2 has a (H3–H4)-binding domain	*Mus musculus, Homo sapiens, Saccharomyces cerevisiae*
	Asf1	Structure analysis and in vitro pull down: H3–H4 dimer bridges the Asf1-Mcm2-7 interaction	*Homo sapiens, Xenopus laevis*
	FACT	In vitro pull down: FACT binds Mcm2 directly	*Homo sapiens, Saccharomyces cerevisiae*

### PCNA: Marking the replication fork for CAF-1-mediated nucleosome assembly

A seminal study in the field of RC nucleosome assembly established that PCNA, the sliding clamp on DNA essential for DNA replication, interacts with the histone chaperone CAF-1 and recruits CAF-1 to the replication fork, thereby marking replicated DNA for nucleosome assembly [92, 93]. PCNA is the sliding clamp for both polymerase δ and polymerase ε during DNA replication in eukaryotes [94, 95]. The crystal structure shows that PCNA is a homotrimer with pseudohexameric symmetry [96]. PCNA is loaded onto the template-primer junction by the clamp-loading RFC complex such that the forward side faces the 3′ end of the primer [97]. Accumulated evidence suggests that PCNA functions as a critical hub protein for recruiting and organizing proteins with different functions during DNA replication, damage repair and many other DNA metabolic events [84, 98]. Fen1 and Cdc9 have been shown to interact with PCNA during Okazaki fragment maturation during replication [99, 100]. Moreover, Dnmt1, a DNA methyltransferase in higher eukaryotes, interacts with PCNA in a replication-coupled mechanism to maintain the DNA methylation pattern during DNA replication [101–103]. Depletion of PCNA inhibited chromatin assembly mediated by human CAF-1, indicating that PCNA is required for CAF-1 to perform nucleosome assembly during chromatin replication in the SV40 system. PCNA colocalizes with CAF-1 during the S phase and directly interacts with CAF-1 [92, 93]. In budding yeast, the mutation of PCNA, which disrupts the PCNA-CAF-1 interaction, leads to a derepression state of the telomeric region and loss of silencing at mating type loci which is similar to the phenotypes of *cac1Δ* [93]. These findings support a model in which PCNA, the sliding clamp of DNA polymerases, recruits CAF-1 to the replication site to perform chromatin assembly activity, thus coupling nucleosome assembly to DNA replication. Notably, PCNA was also found to recruit CAF-1 to DNA damage sites, which is critical for nucleosome assembly during DNA damage repair [104].

Most PCNA-interacting proteins, including yeast CAF-1, bind PCNA through a canonical PCNA-interacting peptide (PIP) and display competitive binding properties [99, 100, 105]. It is worth mentioning that human CAF-1 binds PCNA using two noncanonical PIPs on the p150 subunit, which create relatively weak binding to PCNA compared with the binding through canonical PIPs. It has been proposed that this weak binding to PCNA by CAF-1 may promote nucleosome assembly without disrupting normal DNA replication events [106]. The results from a structural analysis of PCNA mutant proteins with defective gene silencing showed that the mutation sites were all located in a cavity at a distance from canonical binding sites, indicating a second binding mode for the CAF-1-PCNA interaction [107]. PCNA can recruit CAF-1, while CAF-1 itself shows DNA-binding activity. Genetic data showed that the CAF-1 binding capacities for PCNA and DNA were synergistic because mutations in both the WHD domain and PIP domain showed enhanced sensitivity to CPT (camptothecin) and increased loss of gene silencing [108]. A deficiency in new histone supply impairs replication fork rates and leads to the accumulation of PCNA on chromatin [109]. It has been proposed that the retained PCNA provides an opportunity for CAF-1 recruitment to accomplish nucleosome assembly during replication, thus maintaining normal replication rates and genome stability [109]. A deficiency in PCNA unloading leads to loss of the silent state at mating type loci, and this phenotype can be rescued by overexpression of CAF-1, suggesting a role for PCNA unloading in regulating chromatin silencing [110]. It has also been reported that several PCNA mutations disrupting the PCNA-CAF-1 interaction, together with *cac1Δ* showed synergistic defects in the silenced state of the *HMR* region, suggesting that PCNA may contribute to the maintenance of heterochromatin silencing, which is partially independent of the CAF-1-mediated nucleosome assembly pathway [93]. Similar results were observed using the CRASH (Cre-reported altered states of heterochromatin) system, which traced the transient events of silencing loss to the heterochromatin region [111]. It would be interesting to test whether there are other factors that interact with PCNA and that promote RC nucleosome assembly.

### RFC: Potential roles in nucleosome assembly in addition to serving as a sliding clamp loader

The RFC complex functions as a sliding clamp loader and unloader, which is conserved from yeast to human cells [112]. There are three isoforms of RFCs participating in DNA replication, Rfc1-RFC, Ctf18-RFC and the Elg1-RFC complex, which differ in their largest subunits [113]. Ctf18-RFC and Elg1-RFC are also called RFC-like complexes. The Rfc1-RFC complex is the main PCNA loader in vivo*,* and it can also unload PCNA in vitro [114]. However, whether Rfc1-RFC unloads PCNA in vivo remains unclear. The results of the structure analysis of the Rfc1-RFC-PCNA complex showed that RFC binds to the forward side of PCNA in a claw-like manner, drawing PCNA to the 3′ end of the primer by leveraging the ability of PCNA to bind RPA-coated ssDNA [97]. With the hydrolysis of ATP, the PCNA ring closes, and Rfc1-RFC is released from DNA. The function of the Ctf18-RFC-like complex remains poorly understood, and Elg1-RFC is regarded as the main PCNA unloader in vivo [115, 116].

Yeast RFC was found to bind the histone chaperone Asf1 in vitro*,* and the N-terminal of Asf1 contributes to the interaction of Asf1 with Rfc1-RFC [117]. Asf1 was identified as a factor disrupting chromatin silencing when overexpressed in budding yeast [118]. Then, Asf1 was reported as a H3–H4 chaperone acting synergistically with CAF-1 to assemble chromatin [32, 44]. The negatively charged N-terminal of Asf1 contributes to H3–H4 binding, and one Asf1 molecule binds one H3–H4 dimer, disrupting the formation of the H3–H4 tetramer [39, 41, 119, 120]. In addition, Asf1 can deliver the H3–H4 dimer to CAF-1 directly [45]. Asf1 participates in replication-coupled nucleosome assembly by regulating H3K56ac [121], which is crucial to replication-coupled nucleosome assembly and genome stability [27, 37]. The binding of Asf1 to Rtt109 stimulates the activity of the acetylase, leading to the acetylation of the H3 lysine 56 residue [122, 123]. RFC recruits Asf1 to DNA containing a template-primer junction [117]. Deletion of *ASF1* leads to a loss of several replisome components at the stalled fork, including RFC, PCNA and Pol ε, while the association of Pol α at the stalled fork is strengthened [117]. These findings suggest a collaboration between Asf1 and the Rfc1-RFC complex, indicating an impact of nucleosome assembly factors on DNA synthesis, although the function of this interaction remains to be explored.

Recently, it was reported that the Elg1-RFC complex interacts with the histone chaperone Rtt106 and that this interaction is specific for Elg1-RFC. Deletion of *ELG1* leads to enhanced MNase sensitivity and defects in nucleosome assembly at ARS regions [124], indicating a potential role of Elg1-RFC in recruiting Rtt106 to the replication fork, although the function of the Elg1-RFC-Rtt106 interaction remains to be explored. Deletion of *ELG1* leads to an enhanced silence of telomeric regions and affects the maintenance of heterochromatin silencing at mating-type loci [125]. Interestingly, a silencing defect at the mating type region in *elg1Δ* can be rescued by a PCNA-destabilizing mutation or overexpression of CAF-1, indicating that the Elg1-RFC complex regulates chromatin states via the unloading of PCNA [110]. It would be interesting to explore the regulatory role of RFC-histone chaperones in the near future.

### RPA: A platform for histone chaperone coordination

RPA is a conserved ssDNA-binding protein in eukaryotic cells and functions in various DNA transactions [126]. During DNA replication, RPA can coat the ssDNA generated from unwound dsDNA. On the one hand, the binding of RPA protects ssDNA from nuclease digestion, secondary structure formation and lesion creation by damaging agents. On the other hand, the generated RPA-ssDNA complex provides a binding platform with which other factors can interact to coordinate downstream events [126]. RPA contains three subunits named Rfa1, Rfa2 and Rfa3 in budding yeast and RPA70, RPA32 and RPA14 in humans. RPA binds ssDNA with an extremely high affinity, up to 10^−9^ to 10^−10^ M, in a sequence-independent manner [127]. Six OB-folds in the different subunits contribute to the ssDNA-binding ability of RPA. Recently, we found that, in budding yeast, the previously uncharacterized protein Rtt105 functions as an RPA chaperone to facilitate RPA entry into the nucleus and to promote the deposition of RPA onto ssDNA [128, 129]. Cells lacking Rtt105 present a dramatic genome instability phenotype. Remarkably, Rtt105 promotes a fast and stretching mode of RPA binding to ssDNA, suggesting that regulation of the RPA-ssDNA binding platform is crucial for DNA replication [128, 129]. In addition to binding ssDNA, RPA also participates in the regulation of DNA replication. Early studies on the SV40 system showed that the T antigen and RPA together promote the melting of DNA with a viral origin [130]. In a reaction specific to human RPA, the polymerase α-primase complex is recruited by RPA to the melted DNA [131, 132]. RPA can also stimulate the activity of DNA polymerase α [133]. Later, studies using in vitro systems showed that RPA is recruited to the initiation sites after Mcm10 and that mutations in Mcm10 lead to disruptions in RPA recruitment [90, 134]. RPA also regulates the maturation of the Okazaki fragments on the lagging strand according to the length of the flap and the recruitment of nucleases [135, 136].

An early study showed that RPA interacts with the histone chaperone FACT [137]. Recently, we found that RPA can be copurified with not only FACT but also CAF-1 and Rtt106 in budding yeast [138]. Importantly, yeast RPA preferentially binds H3–H4 directly but not H2A–H2B. Moreover, RPA preferentially binds free histones but not nucleosomal histones. Furthermore, preincubating RPA with ssDNA to form an RPA-ssDNA complex promotes the binding of histones with RPA, indicating that RPA binding to histones most likely occurs at the replication fork [138]. Supporting this idea, our biochemical evidence demonstrates that RPA-ssDNA promotes histone H3–H4 assembly immediately on its adjacent dsDNA. Moreover, in the presence of histone H3–H4, the association of RPA with FACT, CAF-1 and Rtt106 is strengthened. Therefore, we proposed that RPA could function as a platform for histone chaperones to connect to the replication fork, thereby promoting the coupling of nucleosome assembly with ongoing DNA replication [138]. Consistent with this idea, it has been reported that human RPA can bind the histone chaperone HIRA and histone H3.3–H4 during gene transcription [139], supporting a role of RPA in DNA replication-independent nucleosome assembly via collaboration with histone chaperones. It would be interesting to determine the regulation of the RPA-ssDNA-binding platform and its potential impact on RC nucleosome assembly.

### DNA polymerases: key players in histone segregation on daughter strands

There are three different DNA polymerases involved in eukaryotic DNA replication. Each new DNA molecule is initiated by the Pol α-primase complex, which synthesizes a short RNA–DNA primer to form a primer-template junction. The resulting primer-template junction is extended by Pol δ and Pol ε to produce the lagging and leading strands [71]. Pol δ and Pol ε possess 3′ to 5′ exonuclease activity, while Pol α lacks this activity [140–142]. The Pol α-primase complex is composed of four subunits: Pol1 (POLA1) and Pol12 (POLA2), which constitute the polymerase part, and Pri1 (PRIM1) and Pri2 (PRIM2), which constitute the primase part. Pol1 and Pri1 are catalytic subunits for DNA synthesis and RNA synthesis, respectively, while the others are regulatory subunits [143]. Pol δ contains three subunits in yeast (Pol3, Pol31 and Pol32) and four subunits (POLD1, POLD2, POLD3 and POLD4) in higher eukaryotes. Pol3/POLD1 is the catalytic subunit. Pol ε contains four subunits: Pol2 (POLE1), which is the catalytic subunit similar to Pol3, and Dpb2 (POLE2), Dpb3 (POLE3) and Dpb4 (POLE4) subunits [144]. Accumulated evidence suggests that Pol ε participates in leading strand synthesis, while Pol δ functions on the lagging strand [145, 146]. Recently, studies monitoring the mutation patterns of daughter strands in DNA polymerase mutants proposed that Pol δ may function on both leading and lagging strands and the Pol ε proofreads the errors generated by Pol δ [147]. In the reconstituted yeast DNA replication system, it was found that DNA polymerases on the leading strand could switch from Pol ε to Pol δ [59, 85]. However, the detailed mechanism of Pol ε and Pol δ on both strands needs to be further explored. The results from a genome-wide analysis of the chromatin binding of DNA polymerase using eSPAN (enrichment and sequencing of protein-associated nascent DNA) established that the chromatin binding of DNA Pol ε has an apparent leading strand bias, while the chromatin binding of both DNA pol δ and Pol displays an apparent lagging strand bias [116].

In yeast, all core histones released from chromatin were detected in the Pol α-associated protein complex [148]. Moreover, H2A–H2B histone-binding sites have been identified in the N-terminal of the large subunit, Pol1, in both yeast and human cells [148]. In yeast, Pol α connects to CMG helicase via the homotrimer hub Ctf4 [149]. Using eSPAN method, it was reported that in wild-type budding yeast cells synchronized in early S phase by HU treatment, H3K56ac, a mark of newly generated histones, displays a slight leading strand bias, and H3K4me3, a parental mark, displays a slight lagging strand bias [150]. The eSPAN method results showed that disrupting the interaction of Pol α and Ctf4 led to a leading strand bias for parental histone marker H3K4me3 distribution, indicating that the Mcm2-Ctf4-Pol α pathway is important for recycling the parental histones and depositing them onto the lagging strand [150]. It is worth mentioning that the mutation that abolished the histone-binding activity of Mcm2 disrupted the association between Pol α and Mcm2 without affecting the interaction of Pol α with Ctf4. Disrupting the H2A-H2B-binding site of Pol α led to similar results [148]. Early studies also showed that Pol α can bind FACT [151–153]. Intriguingly, the amount of FACT complex copurified with Pol α increased in the absence of Ctf4 [152]. Moreover, the interaction of Pol α with FACT was reduced by disrupting the H2A–H2B-binding activity of Pol α [148]. It would be interesting to decipher the relationships among Mcm2, Ctf4, Polα, FACT and histones and to determine how these interactions are involved in parental histone H3–H4 recycling.

An early proteomic study showed that all four core histones copurified with the Pol ε complex in budding yeast [154]. Recently, it was reported that the two subunits of Pol ε, POLE3 and POLE4, which each possess a H2A–H2B histone fold motif, formed a stable dimer and could bind histone H3–H4 directly but not H2A-H2B in mammalian cells [155–157]. POLE3-POLE4 could not only bind H3-H4 dimers and tetramers but also promote the deposition of H3–H4 tetramers onto DNA directly. Transient depletion of POLE3-POLE4 affects both the deposition of newly synthesized histones and the recycling of parental histones. Thus, POLE3-POLE4 has a role in chaperoning histone H3–H4 [155]. Interestingly, the eSPAN analysis results showed that deletion of *DPB3* or *DPB4* leads to a dramatic increase in lagging strand bias for parental histones, indicating a pivotal role of Dpb3 and Dpb4 in recycling parental histones and depositing them onto leading strands [158].

Taken together, both Pol ε and the Pol α complex showed histone-binding activity. Moreover, disrupting the histone-binding ability of either Pol ε or Pol α impaired parental histone segregation on daughter strands, indicating a direct role of polymerase in RC nucleosome assembly. Supporting this idea, mutations disrupting the Pol α-H2A–H2B interaction and the Pol α-Ctf4 interaction led to silencing loss in telomeric regions and at mating type loci [148]. Pol α was also important for heterochromatin silencing maintenance in fission yeast and the restoration of repressive histone marks in plants [159, 160]. Similarly, it has been shown that both the Pol ε catalytic subunit Pol2 and the regulatory subunits Dpb3-Dpb4 are important for the maintenance of epigenetic states in subtelomeric regions and heterochromatin boundary specificity in budding yeast [161, 162]. *dpb3Δ* and *dpb4Δ* cells also showed silencing loss at mating-type loci [158]. It has also been reported that Dpb3 and Dpb4 are important for maintaining the silent state of heterochromatin in fission yeast [163]. It would be interesting to determine whether Pol δ has histone-binding activity and its functions in regulating histone segregation on daughter strands. Furthermore, it has been reported that both Pol ε and Pol α in budding yeast are copurified with the FACT complex [57]. However, the functions of these interactions are not yet understood. It would be interesting to dissect the collaboration between FACT and polymerases during RC nucleosome assembly.

### Mcm2: Vanguard of nucleosome disassembly and parental histone recycling

Stimulated when cells enter the S phase, the activated helicase MCM2-7 starts to unwind and move along DNA. Nucleosomes ahead of the replication fork act as barriers for the replication machinery and must be disassembled to allow the fork to pass. The MCM2-7 complex, the leading replisome protein, has the highest likelihood of contacting parental nucleosomes. It has been shown that the MCM2-7 complex can bind H3 and H4 in HeLa cell extracts [164]. Subsequent studies in mouse cells demonstrated that the Mcm2 subunit binds H3–H4 and assembles the nucleosome-like structure in vitro*,* suggesting potential chaperone activity for Mcm2 [165]. Moreover, a histone-binding domain (HBD) was identified in the N-terminus of mouse Mcm2, and structural analysis results indicated that two N-terminal domains can bind H3-H4 tetramers and hijack DNA-binding sites in intact nucleosomes [165–167]. These studies increase the likelihood that Mcm2 is involved in the disassembly of histone H3–H4 tetramers on parental nucleosomes. Consistent with this supposition, yeast Mcm2 also has an HBD that associates with all four histones released from chromatin [57].

Recently, two reports showed that Mcm2 directly regulates histone segregation on daughter strands[150, 168]. Interestingly, the Mcm2-3A mutation, which disrupts the interaction between Mcm2 and histones, results in an apparent lagging strand bias for H3K56ac and an apparent leading strand bias for H3K4me3. Similarly, a method called SCAR-seq (sister chromatids after replication by DNA sequencing) was developed to map the distribution of both parental histones (i.e., H4K20me2) and new histones (i.e., H4K5ac) on daughter strands in mouse ES cells [168]. At active replication forks, the segregation of parental histones was almost symmetrical, although a slight bias to the leading strand was shown. Mcm2-2A, the mutation disrupting histone binding to Mcm2, resulted in leading strand bias for parental histone mark such as H4K20me2 and a lagging bias for new histone mark such as H4K5ac. Together, these findings demonstrated that the histone-binding activity of Mcm2 promotes the transfer of parental histones to the lagging strand.

In addition to histones, the MCM2-7 complex was reported to be copurified with histone chaperones such as Asf1 and the FACT complex [57, 166, 169–174]. In mammalian cells, the interaction of Asf1 with the MCM2-7 complex occurs in the nucleus, forming a bridge with histone H3–H4 [169]. The *asf1-V94R* mutant, which was shown to disrupt the binding of H3–H4 to Asf1, failed to bind Mcm2 [169]. Moreover, *asf1Δ* led to impaired DNA unwinding, and the purified histones separated from this complex lacked marks on newly synthesized histones. Furthermore, the Asf1-H3-H4-Mcm2 complex accumulated upon HU treatment, indicating that this complex is a possible intermediate in parental histone recycling. The structure of the Asf1-H3-H4-Mcm2 complex was determined and supported this notion [166, 167, 170, 172]. In addition, Asf1 and Mcm2 were found to function together to cochaperone H3-H4 dimers and assemble them on DNA [166, 167, 170]. Similar coordination was also discovered using *Xenopus* egg extracts [175]. The histone chaperone Asf1 was shown to facilitate the removal of nucleosomes at promoter regions or gene bodies during transcription [176–178]. However, Asf1 could not split the H3-H4 tetramer into dimers directly in vitro*,* suggesting that other factors must cooperate with Asf1 to accomplish histone separation in vivo [179]. Together, these studies established that Mcm2 can function as a histone chaperone and participates in parental histone recycling. Notably, the histone H3-H4 dimer exists in the Asf1-H3-H4-Mcm2 complex. It would be interesting to test whether the tetramer splits apart during DNA replication.

The FACT complex is another histone chaperone that was copurified with MCM2-7 [57, 148, 173, 174]. FACT mainly contains two subunits, Spt16 and Pob3 (SSRP1 in mammalian cells), and a free HMGB (High mobility group-box) subunit called Nhp6 [180]. FACT was previously shown to promote transcription elongation by facilitating RNA polymerase II movement across the nucleosomal template in vitro [52, 181]. When RNA Pol II approaches a nucleosome, the movement of Pol II leads to the partial uncoiling of nucleosomal DNA, which is called nucleosome reorganization [182, 183]. FACT can remove one H2A-H2B dimer during reorganization and deposit it onto the H3-H4 tetramer or nucleosome hexamer in vitro [184, 185]*.* A recent study demonstrated that FACT itself could not disassemble nucleosomes in vitro [186]. Moreover, DNA replication initiation was delayed when the FACT-MCM2-7 interaction was disrupted [173]. FACT promoted DNA unwinding by MCMs and fork progression on the nucleosomal template in vitro*,* which suggested that FACT could function together with MCMs to facilitate nucleosome disassembly [59, 173]. Other studies showed that FACT could maintain the replication fork rates and promote the progression of the S phase in vivo [174, 187]. In addition, FACT interacts with the MCM2-7 complex when histones are released from chromatin in cell extracts, suggesting that this interaction occurs after the nucleosomes are disassembled [57]. Several amino acids in the histone-binding domain of Mcm2 are essential for the interaction of FACT and MCM, indicating that histones were also bridged during this interaction. Disruption of the histone-binding activity of Mcm2 led to loss of silencing in the subtelomeric region, indicating that the chromatin states had been altered [57]. These findings illustrate that FACT functions together with MCM2-7 during replication to regulate nucleosome disassembly, thus maintaining the chromatin state.

## Conclusion

**Fig. 1:**
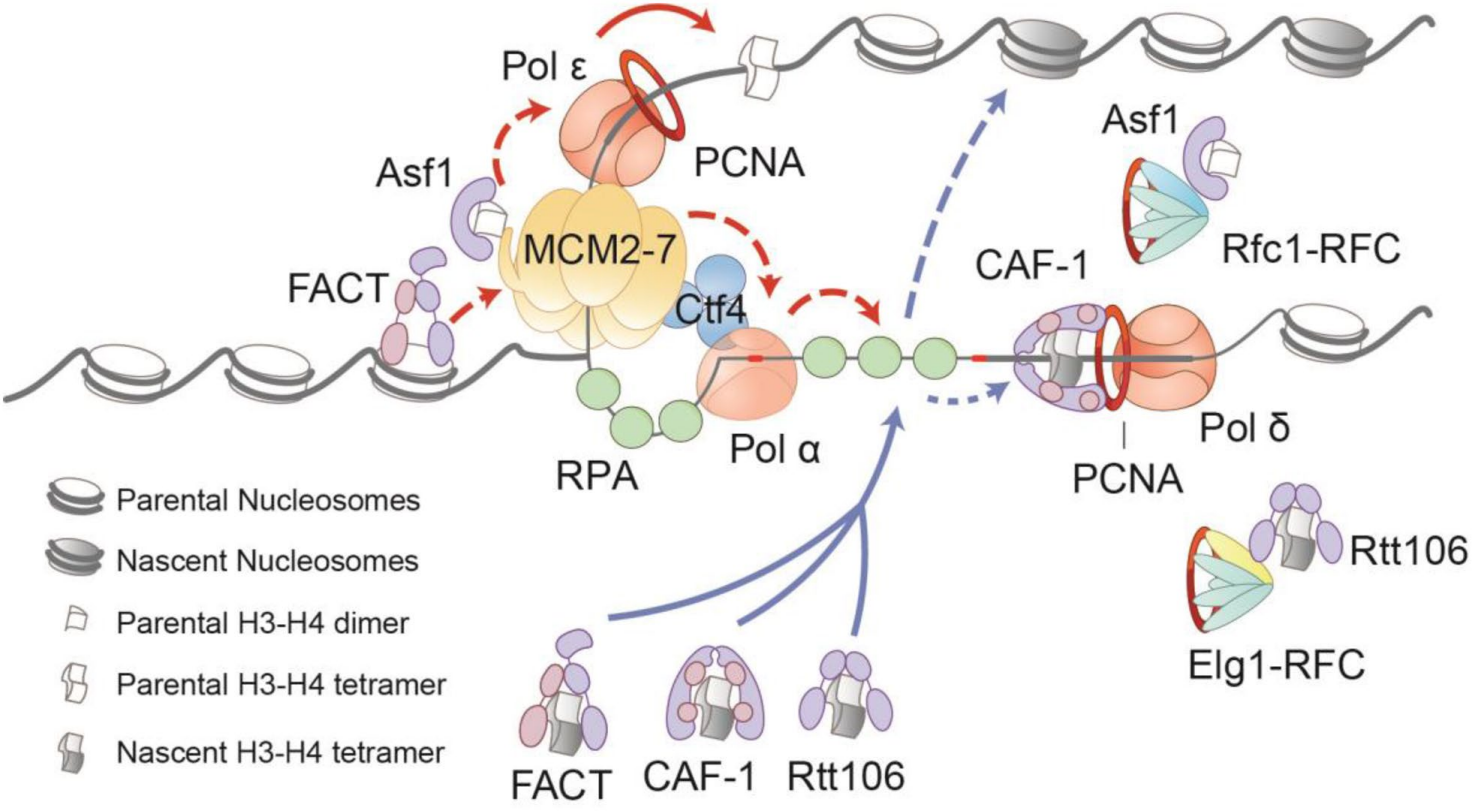
The replisome provides a guide to modulate DNA replication-coupled nucleosome disassembly and assembly. When the replication fork is moving forward, the replicative helicase Mcm2 might cooperate with the histone chaperone Asf1 or FACT to facilitate the disassembly of parental nucleosomes and the recycling of parental histones. Dpb3-Dpb4 (POLE3-POLE4), the subunits of DNA Pol, provide a guide for the assembly of parental histones on the leading strand. The Mcm2-Ctf4-Pol α axis directs the recycling of the parental histone to the lagging strand. RPA-ssDNA functions as a general platform that directs the histone chaperones (CAF-1, Rtt106 and FACT) carrying newly synthesized histones to enter the replication fork, thereby modulating nucleosome assembly on daughter strands. PCNA marks nascent DNA and promotes CAF-1-mediated nucleosome assembly. In intriguing findings, the main PCNA loader Rfc1-RFC interacts with Asf1, and the PCNA unloader Elg1-RFC interacts with Rtt106

In summary, recent advances have illuminated the contribution of the replisome to RC nucleosome assembly (Fig. 1). First, several replisome components guide histone chaperones to connect with the replication fork. For instance, PCNA marks nascent DNA, guiding CAF-1-mediated nucleosome assembly. When the replicative helicase approaches the parental nucleosome, Mcm2 might guide the disassembly of the parental nucleosome and recycle parental histones in collaboration with histone chaperone Asf1 or FACT. Second, RPA serves as a general platform to promote RC nucleosome assembly. RPA binds almost all histone chaperones connected to replication forks, including CAF-1, Rtt106 and FACT. RPA binds both parental histones and new histones. It would be interesting to test the regulation of this RPA-ssDNA platform in the future. Third, replisome components regulate histone segregation on daughter strands. It has been established that the Mcm2-Ctf4-Pol α axis transfers parental histones onto the lagging strand, while Pol ε regulates the recycling of parental histones, depositing them on the leading strand. Considering that some replisome components, such as Mcm2 and Pol α, have histone-binding motifs, it would be interesting to test the intrinsic chaperone activities of these replisome proteins directly and to determine the cooperative mechanisms with histone chaperones. Therefore, the replisome provides a guide and modulates nucleosome assembly during DNA replication.

Recently, it was reported that parental histones are redeposited locally in repressed chromatin domains [188]. As a potential carrier of epigenetic information, the recycling of parental histones after the passage of the replication fork is important for the inheritance of chromatin states. Over the past decades, studies by many investigators have suggested that many RC nucleosome assembly factors impact heterochromatin silencing. Consistent with this finding, mutations of the replisome component genes, such as *mcm2-3A*, *pol1-4A*, or *pol30-879*, lead to the loss of histone-binding activity or histone chaperone-binding activity, often resulting in loss of heterochromatin silencing [57, 93, 148]. Intriguingly, the degree of loss of silencing in replisome component mutants was often lower compared with that induced by mutations at histone modification sites or in nucleosome assembly factors [189, 190]. More recently, structure studies using Cryo-EM techniques have revealed extensive details on the cooperation among replisome components, especially the cooperation among the replicative helicase and DNA polymerases [87, 191]. This evidence strongly supports a model of replisome components that directly contribute to histone dynamics. Considering the importance of the inheritance of heterochromatin in preserving cell identity, dissecting the regulatory role of replisome components during RC nucleosome assembly is fundamentally important.

## Data Availability

Not applicable.

## Abbreviations

ARS: autonomously replicating sequence
SILAC: stable isotope labeling by amino acids in cell culture
PIP: PCNA-interacting peptide
eSPAN: enrichment and sequencing of protein-associated nascent DNA
SCAR-seq: sister chromatids after replication by DNA sequencing
IP: immunoprecipitation
CRASH: cre-reported altered states of heterochromatin
